# Supplementary material for: Genome-wide CRISPR Screens in T Helper Cells Reveal Pervasive Crosstalk between Activation and Differentiation
Source: Cell. 2019 Feb 7;176(4):882–896.e18. doi: 10.1016/j.cell.2018.11.044 (PMC6370901; doi:10.1016/j.cell.2018.11.044)
Supplement: Data S2. Processed Data from All the Steps of the Analysis, Related to Figure 1 [file mmc2.zip › supplemental data/motif analysis/Bhlhe40_homer/homerResults/motif2.similar.html]

motif2

## Information for motif2

T
C
G
A
A
C
G
T
A
C
T
G
C
G
T
A
T
A
G
C
C
G
A
T
T
G
A
C
G
T
C
A
  
Reverse Opposite:  

A
C
G
T
A
C
T
G
G
C
T
A
A
T
C
G
A
C
G
T
G
T
A
C
C
G
T
A
A
G
C
T
  

|  |  |
| --- | --- |
| p-value: | 1e-39 |
| log p-value: | -9.086e+01 |
| Information Content per bp: | 1.829 |
| Number of Target Sequences with motif | 122.0 |
| Percentage of Target Sequences with motif | 10.17% |
| Number of Background Sequences with motif | 1009.3 |
| Percentage of Background Sequences with motif | 2.37% |
| Average Position of motif in Targets | 110.0 +/- 50.9bp |
| Average Position of motif in Background | 96.8 +/- 68.3bp |
| Strand Bias (log2 ratio + to - strand density) | 0.0 |
| Multiplicity (# of sites on avg that occur together) | 1.09 |
| Motif File: | file (matrix) reverse opposite |

### Similar de novo motifs found

|  |  |  |  |  |  |  |  |
| --- | --- | --- | --- | --- | --- | --- | --- |
| Rank | Match Score | Redundant Motif | P-value | log P-value | % of Targets | % of Background | Motif file |
| 1 | 0.965 | A G C T T C A G A C G T A C T G C G T A A T G C A C G T G T A C C G T A A G C T | 1e-37 | -86.176215 | 10.83% | 2.81% | motif file (matrix) |
| 2 | 0.946 | C T G A A G T C C A G T T A C G A C G T A T C G C G T A A T G C G C A T A G T C C G T A A G T C | 1e-28 | -66.409830 | 11.08% | 3.60% | motif file (matrix) |
| 3 | 0.741 | A T G C G C A T G A C T C G T A A C T G G C A T A G T C C G T A G C A T T C G A A G C T G T A C | 1e-15 | -36.816049 | 1.17% | 0.04% | motif file (matrix) |
| 4 | 0.656 | A C G T C G T A G T A C C T A G C A G T G T A C T G C A A T G C | 1e-11 | -26.318499 | 7.75% | 3.52% | motif file (matrix) |
